# Alpha-D-glucan nanoparticulate adjuvant induces a transient inflammatory response at the injection site and targets antigen to migratory dendritic cells

**DOI:** 10.1038/s41541-017-0007-7

**Published:** 2017-02-23

**Authors:** Fangjia Lu, Yung-Yi C. Mosley, Randol J. Rodriguez Rosales, Brooke E. Carmichael, Srikanth Elesela, Yuan Yao, Harm HogenEsch

**Affiliations:** 1 Department of Comparative Pathobiology, College of Veterinary Medicine, Purdue University, 725 Harrison Street, West Lafayette, IN 47907 USA; 2 Department of Food Science, College of Agriculture, Purdue University, West Lafayette, IN 47907 USA

## Abstract

Biodegradable nanoparticles with functionalized surfaces are attractive candidates as vaccine adjuvants. Nano-11 are cationic dendrimer-like *α*-D-glucan nanoparticles with a diameter of 70–80 nm. Mice injected with antigen formulated with Nano-11 developed antibody titers that were similar or greater than antigen with aluminum adjuvant. Utilizing an in vivo imaging system, Nano-11 was shown to remain at the injection site after administration and cleared gradually over the course of 3 weeks. Injection of Nano-11 induced a transient inflammatory response characterized by recruitment of a mixed population of inflammatory cells, predominantly monocytes and macrophages with relatively few neutrophils. Recruited Mac-2^+^macrophages efficiently phagocytized the majority of Nano-11 at the injection site. Fluorescently labeled Nano-11 was present in cells in the draining lymph nodes 1 day after injection, with the majority contained in migratory dendritic cells. Injection of ovalbumin adsorbed to Nano-11 resulted in an increase of ovalbumin-containing cells in draining lymph nodes. Nano-11 delivered more antigen to antigen-presenting cells on a per cell basis and demonstrated more specific targeting to highly immunopotentiating migratory dendritic cells compared with soluble or aluminum hydroxide adsorbed ovalbumin. These results support the efficacy of Nano-11 and its potential use as a next generation vaccine adjuvant.

## Adjuvants: Discovering the next generation of vaccine boosters

A plant-derived nanoparticle boosts the immune system’s response to foreign stimuli, such as vaccines, through newly discovered mechanisms. Harm HogenEsch and his team from Purdue University, USA, previously discovered that their formulated nanoparticle adjuvant, Nano-11, increased immune responses to stimuli in mice. Adjuvants are formulations often co-administered with vaccines to promote interaction with the host’s immune system, conferring greater protection. In this study, the scientists found that Nano-11 functions by inducing a temporary inflammation, attracting immune cells to the injection site. The nanoparticles also facilitate the transport of the stimuli to the lymph nodes. Nano-11 is metabolized without deposition in any major internal organs, suggesting a positive safety profile. As current aluminum-based adjuvants are sometimes poorly effective and can cause local adverse reactions, nanoparticle-based potentiators such as Nano-11 may form the next generation of adjuvants.

## Introduction

Modern vaccines are increasingly formulated with antigens consisting of subunits of microbial pathogens to enable a more focused immune response while increasing the safety compared with whole killed and attenuated pathogens.^1^ These subunit vaccines often require adjuvants in order to stimulate an effective immune reaction.^2–4^ Human vaccines are usually formulated with aluminum-containing adjuvants. Although they have an excellent safety record and are generally effective at inducing an antibody response, aluminum adjuvants have several drawbacks including poor stimulation of cell-mediated immune responses, susceptibility to freezing, and occasionally adverse reactions at the injection site.^5, 6^ Veterinary vaccines often contain adjuvants other than aluminum adjuvants including water-in-oil emulsions and saponins, but these are often associated with significant local reactions at the injection site. Thus, there is a need for new safer and more effective adjuvants to protect both human and animal populations.

Nanoparticles refer to particulate materials that are less than one micrometer in diameter.^7^ Similar to many intracellular pathogens, they can be readily taken up by immune cells making them good candidates as vaccine adjuvants.^8^ Indeed, the very first reported medical application of nanoparticles, published in 1976, was the use of polymeric micelle nanoparticles as adjuvant.^9^ The ability to alter the size, shape, and surface of nanoparticles has stimulated great interest in nanotechnology to design the next generation of adjuvant.^7, 10, 11^

Nanoparticles can boost the immune response in multiple ways. They can induce inflammatory reaction at the injection site, which recruits immune cells to the proximity of antigens, a phenomenon termed “reverse targeting”.^4^ Through encapsulation or adsorption of antigens on their surface, nanoparticles can enhance the uptake of antigen by antigen-presenting cells (APCs). The antigen-containing APCs migrate to the draining lymph node to activate antigen-specific T cells and drive the adaptive immune response. Alternatively, some nanoparticles drain via the lymph to the lymph node, where they can be picked up by lymphoid tissue resident dendritic cells (DCs).^12^ Clearance of nanoparticles is a concern for medical applications, and wide tissue distribution and long-term deposition following injection of these particles are not desirable. Phagocytic cells recruited to the injection site can phagocytize and subsequently digest the nanoparticles if they are biodegradable. Alternatively, nanoparticles may be excreted via the kidneys or liver.^13^

Phytoglycogen (PG) nanoparticles are polysaccharide structures derived from plants. They exist in large quantities in the kernel of a genetic variant of sweet corn, *sugary-1*. The deficiency of a starch debranching enzyme results in the formation of highly branched, dendrimer-like PG nanoparticles that replace starch granules.^14, 15^ The surface of PG nanoparticles can be chemically modified to give them different chemical and biological properties. We recently reported that a functionalized PG nanoparticle 70–80 nm in diameter with positive charge and an amphophilic surface, termed Nano-11, enhanced the delivery of absorbed antigen to DCs and subsequently activated DCs in vitro.^16^ Formulation of Nano-11 with different antigens significantly increased the immune response, demonstrating its adjuvant effect. However, the distribution and fate of Nano-11 after injection are unknown. Here, we investigated the mechanism of Nano-11 clearance after administration, its interaction with immune cells in vivo, and how it affects the delivery of coinjected antigen to the draining lymph node.

## Results

### Nano-11 has comparable adjuvanticity as aluminum hydroxide adjuvant

**Fig. 1:**
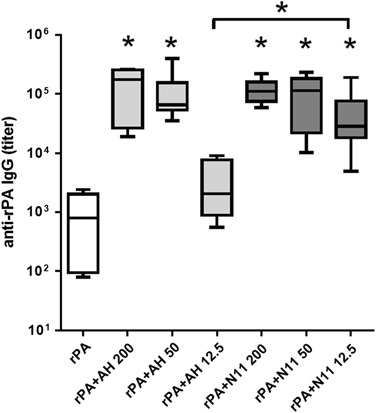
Nano-11 has comparable adjuvanticity as aluminum hydroxide adjuvant (AH) and shows superior immunopotentiation at low dose. Mice were immunized twice with 2 μg of recombinant protective antigen (rPA) from *Bacillus anthracis* alone or adjuvanted with either AH or Nano-11 at three different dosages (200, 50, and 12.5 μg). Titers of rPA-specific IgG in the serum were measured through ELISA. Mean ± SEM of six mice. *p < 0.05

We recently demonstrated that Nano-11 significantly enhanced the immune response toward protein antigens.^16^ To compare the immunostimulatory effect of Nano-11 with that of aluminum hydroxide adjuvant (AH) mice were injected with recombinant protective antigen (rPA) from *Bacillus anthracis* with different doses of Nano-11 or AH. Nano-11 and AH significantly enhanced the titer of rPA-specific IgG in the serum to the same level when used at 200 and 50 µg (Fig. 1). However, at a low dose of 12.5 μg Nano-11 still demonstrated good adjuvanticity, similar to high and medium doses, while AH failed to stimulate the immune response. The majority of IgG antibodies were IgG1 with low titers of IgG2a suggesting that both adjuvants support primarily a Th2 response (Suppl. Fig. 1). This indicates that Nano-11 had a comparable immunopotentiating effect as the widely used AH adjuvant and performed better than AH at low doses when combined with rPA.

### Nano-11 does not disperse after injection and is cleared from the injection site over time

**Fig. 2:**
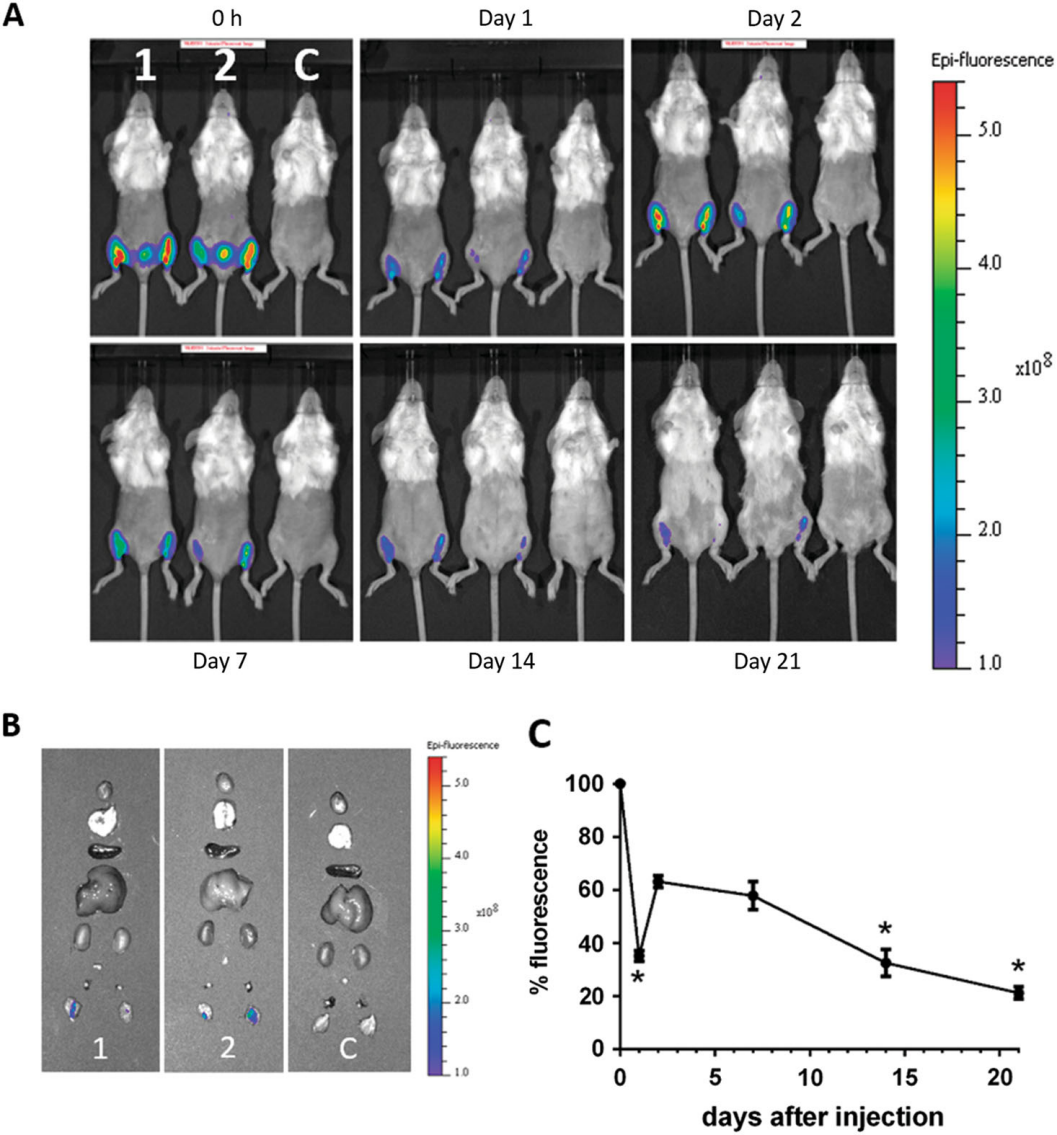
Nano-11 is retained at the injection site and cleared with time. **a** Mice were injected in both calf muscles with 200 µg Alexa Fluor 647-labeled Nano-11 (1 and 2) or nonlabeled Nano-11 (control) with 10 µg OVA and imaged under a fluorescence imager at different time points. **b** Internal organs (from *top* to *bottom*: heart, lung, spleen, liver, kidneys, iliac lymph node, popliteal lymph nodes, and injection sites) of mice were harvested on day 21 and imaged. **c** Fluorescence intensity of the injection site over time expressed as percentage of the intensity at 0 h. Mean ± SEM of five mice (days 0, 1, 2, 7) or three mice (days 14 and 21). *Significantly different from day 2 fluorescence (*p* < 0.001)

A whole mouse imaging system was used to study the distribution of Nano-11 after injection over a 3-week period. Following injection of Alexa Fluor 647-labeled Nano-11 into the calf muscles of both legs, fluorescent signal could be readily detected at the injection site (Fig. 2a). The signal was confined to the injection site and its intensity gradually decreased over the observation period. To confirm that the injected nanoparticles did not distribute to internal organs, major organs from mice were excised at the end of the observation period and examined under the imager. Fluorescent signal was only detected at the injection sites and not in internal organs (Fig. 2a). Imaging of dissected tissues harvested on days 1 and 7 revealed low fluorescence signals in the iliac lymph nodes in addition to fluorescence in the injection sites (Suppl. Fig. 2). Quantification of the fluorescence intensity at different time points after immunization showed that the fluorescent signal was strongest when labeled Nano-11 was first injected into the muscle, rapidly decreased on day 1 before a moderate, but significant (*p* < 0.001), increase on day 2. After day 7, the fluorescent signal steadily decreased over time and by day 21 only about 20% of the original signal could be detected (Fig. 2c).

### Nano-11 induces a transient inflammatory response at the injection site

**Fig. 3:**
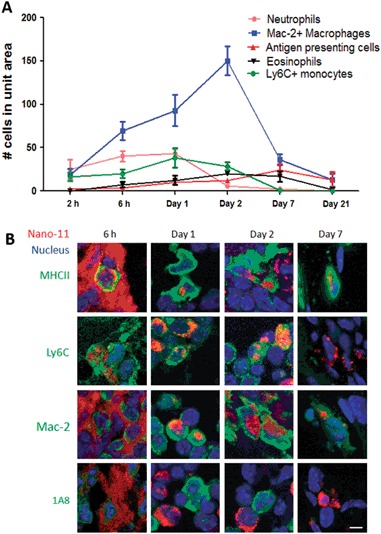
Nano-11 induces transient inflammation at the injection site and is taken up by recruited cells. **a** Injection sites were collected at different time points after intramuscular injection of 200 µg Nano-11 combined with 10 µg ovalbumin. Cells were identified with monoclonal antibodies and phenol red (converted by eosinophil peroxidase into red fluorescent product) and their number was counted under a fluorescent microscope. The data indicate the number of cells per high-power field and represent mean ± SEM of four samples. **b** Mice were injected with 200 µg Alexa Fluor 647-labeled Nano-11 with 10 µg OVA and tissue samples were labeled as indicated. Confocal images were collected with a 60× objective lens and are representative of two independent experiments. Bar=10 µm

Inflammation induced at the site of vaccination plays an important role in the initiation of the immune response, but excessive or persistent inflammation may lead to local adverse reactions. The severity and type of inflammation after injection of 200 µg Nano-11 with 10 µg ovalbumin (OVA) were investigated at different time points after injection (Fig. 3a). Inflammatory cells appeared at the injection site starting from 2 h after immunization and their number rapidly increased until day 2. After day 2, the number of inflammatory cells declined and few cells remained by day 21. Mac-2^+^ inflammatory macrophages comprised the majority of inflammatory cells. A moderate number of neutrophils were present at 6 h and their number dropped sharply after day 1. A few APCs (MHCII^+^) and eosinophils first appeared at 6 h and their numbers gradually increased until day 7. Monocytes (Ly6C^+^) appeared early at the injection site and their number decreased by day 7. The relative abundance of Mac-2^+^ cells contrasted with the injection sites of aluminum-containing adjuvants in which neutrophils were the most abundant cells on days 1 and 2 after immunization (Suppl. Fig. 3).

### Nano-11 is taken up by APCs, macrophages, and monocytes

To determine which types of cells take up Nano-11, 200 µg Alexa Fluor 647-conjugated Nano-11 with 10 µg OVA was injected into mice andinjection sites were collected at 6 h, day 1, day 2, and day 7 (Fig. 3b). At 6 h, Nano-11 was mainly extracellular, but on day 1 most of the red fluorescence was intracellular indicating active phagocytosis of Nano-11. Nano-11 was present in MHC II^+^ APCs at all time points examined. Nano-11 was also found inside Ly6C^+^cells at 6 h, day 1, and day 2. The number of Ly6C^+^ cells was very small on day 7, and none of them contained Nano-11. Mac-2^+^cells with intracellular Nano-11 were first observed on day 1. By day 2, Nano-11 was largely contained in Mac-2^+^ cells (Suppl. Fig. 4). There was no evidence of uptake of Nano-11 by neutrophils at any time point. Nano-11 was also not detected in muscle cells or fibroblasts at the site of injection.

### Nano-11 is transported mainly by migratory DCs to the draining lymph node

Antigens reach draining lymph nodes after uptake by migratory DCs (MigDCs) or by diffusion in lymph followed by uptake of lymph node resident DCs. To determine the type of cells that contained Nano-11 in the draining lymph nodes, mice were injected with Alexa Fluor 647-conjugated Nano-11 and OVA, and draining (iliac) lymph nodes were harvested at various time points. Among the myeloid cells (identified as FSC^medium−high^ and SSC^medium−high^), Nano-11-containing cells were differentiated into three subpopulations (Suppl. Fig. 5). MigDCs were identified as MHCII^high^ and CD11c^medium^ cells and lymphoid tissue resident DCs (LTDCs) as MHCII^medium^ and CD11c^high^ cells.^17^ The third population was MHCII^low−medium^ and CD11c^low^ and these were mainly Ly-6C^+^ monocytes and F4/80^+^ macrophages (Suppl. Fig. 6).

**Fig. 4:**
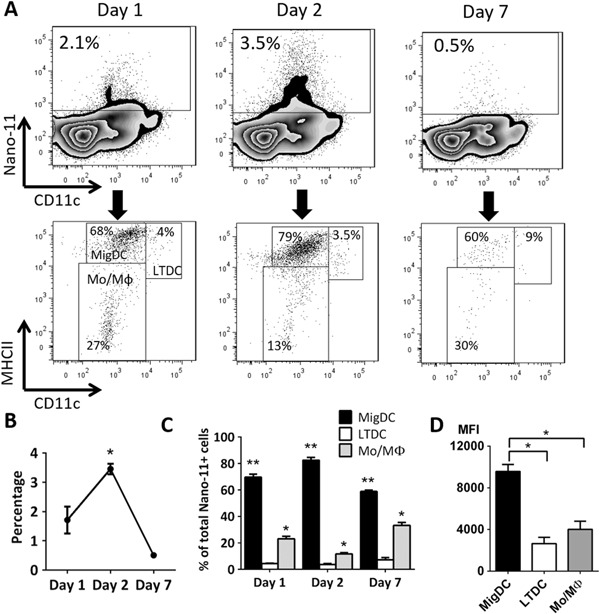
Nano-11 is actively transported by APCs to the draining lymph node. **a** Detection and phenotyping of Nano-11-containing myeloid cells at different time points in the draining lymph node. Data are representative of three independent experiments. **b** Percentage of Nano-11-containing cells among all myeloid cells in the draining lymph node over time. Mean ± SEM of three mice; *significantly different from day 1 and day 7 (*p* < 0.05) **c** Distribution of Nano-11 in different subtypes of APCs in the draining lymph node over time. Mean ± SEM of three mice; * and ** significantly different from the other two groups (*p* < 0.01). **d** Uptake of Nano-11 per cell for each APC subtype as indicated by the mean fluorescence intensity (MFI). Mean ± SEM of three mice; *p < 0.01

Cells containing Nano-11 were detected throughout the first 7 days after immunization, with the highest amount of Nano-11 detected on day 2 when >3% of all myeloid cells in the lymph node were Nano-11^+^ (Fig. 4a, b). The majority of Nano-11-positive cells (60–80%) were MigDC. Macrophages and monocytes comprised 10–30% of the Nano-11-positive cells and <10% of Nano-11 was in LTDCs (Fig. 4c). The mean fluorescence intensity (MFI) for AF647-labeled Nano-11 in MigDCs was about twice that in macrophages/monocytes and LTDCs indicating that these cells contained more Nano-11 on a per cell basis (Fig. 4d).The small population of neutrophils and eosinophils in the draining lymph node did not contain Nano-11 (Suppl. Fig. 7).

### Nano-11 facilitates targeting of adsorbed antigen to MigDCs and enhances its transportation to the draining lymph node

**Fig. 5:**
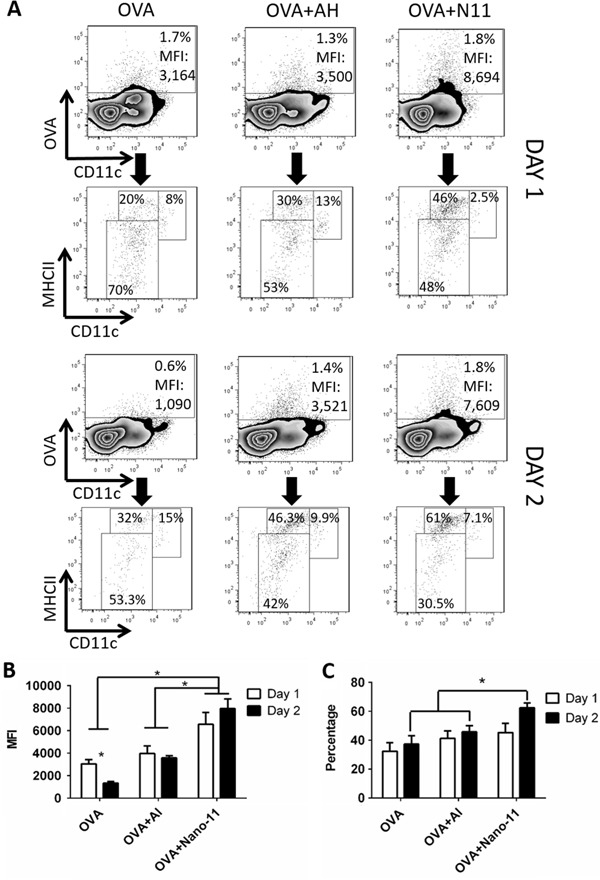
Nano-11 facilitates the transport of antigens to lymph nodes. **a** Detection and phenotyping of OVA^+^ cells in the draining lymph nodes of mice injected with 10 µg Alexa Fluor 647-conjugated OVA alone or adjuvanted with either 200 μg AH or 200 μg Nano-11 on day 1 and day 2. Data are representative of experiments with six mice. **b** Mean fluorescence intensity of OVA in OVA-containing cells in the draining lymph node on day 1 and day 2 with mice injected with different formulations. Bars represent the mean ± SEM of six mice. *p < 0.05. **c** Percentage of MigDCs among OVA-positive cells in the draining lymph nodes of mice injected with different formulations. Bars represent the mean ± SEM of six mice. *p < 0.05

Negatively charged antigens adsorbed onto the surface of Nano-11 and this enhanced uptake of antigen by DCs in vitro.^16^ To determine if Nano-11 also facilitated the transport of antigen to the draining lymph node in vivo, Alexa Fluor-647 labeled OVA alone or combined with Nano-11 was injected into mice and iliac lymph nodes were harvested on day 1 and day 2 after immunization. For comparison, labeled OVA was adsorbed to AH. Previous studies have demonstrated that AH increased antigen uptake by DCs in vitro.^18^ On day 1 after immunization, around 1.5% of myeloid cells in the draining lymph node contained OVA for all three formulations (Fig. 5a). On day 2 after immunization, the percentage of OVA-containing myeloid cells in the lymph node dropped sharply to about 0.5% in mice injected with OVA only, while for both Nano-11 and AH-adjuvanted vaccines, the percentage of OVA containing myeloid cells remained at around 1.5% (Fig. 5a). Although OVA was present in about the same percentage of cells in the lymph node for all three formulations on day 1, the MFI of OVA in cells was almost twice as high when OVA was mixed with Nano-11 compared with the other two formulations (Fig. 5b), suggesting Nano-11 enhanced the delivery of antigen to APCs on a per cell basis. The MFI was much lower for mice injected with antigen only on day 2, but it remained high for adjuvant-containing formulations with a significantly higher MFI for theNano-11 formulation compared with AH (Fig. 5b). In addition, Nano-11 increased the uptake of OVA by MigDCs compared with soluble OVA and AH-adsorbed OVA (Fig. 5c).

**Fig. 6:**
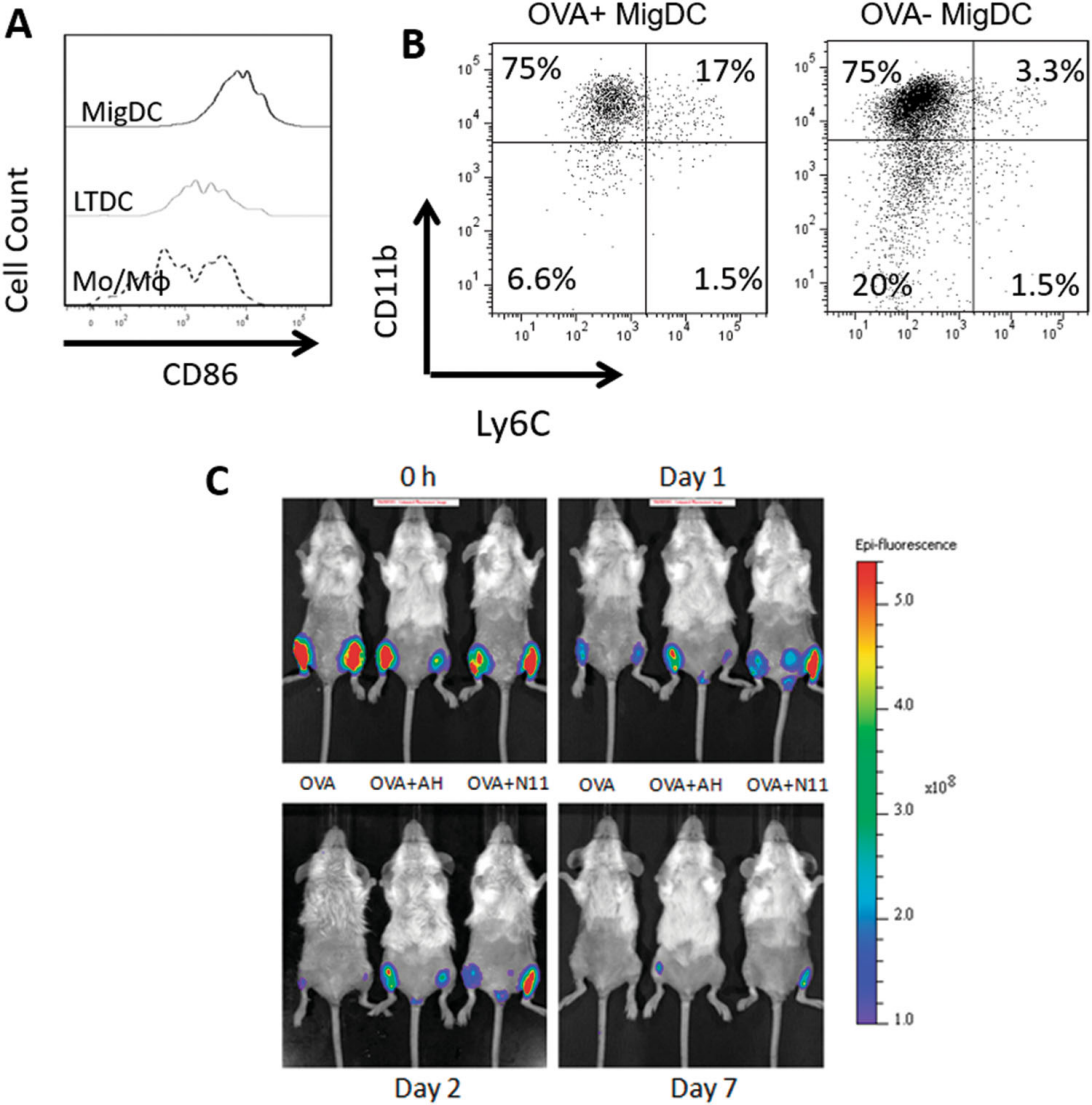
Nano-11 improves antigen targeting to immunopotentiating cells in the draining lymph node and increases retention of antigen at the injection site. **a** Expression of CD86 on different types of OVA^+^ cells in the lymph node. **b** Phenotyping of OVA^+^ and OVA^−^ MigDCs in the draining lymph node. Lymph nodes were harvested 2 days after injection of 10 μg Alexa Fluor 647-labeled OVA adjuvanted with 200 μg Nano-11 and data are representative of two independent experiments. **c** Mice were injected with 10 μg of Alexa Fluor 647-conjugated OVA alone or adjuvanted with either AH or Nano-11 and examined with a fluorescence imager over time. Data are representative of two independent experiments

In order to function as effective APCs, DCs need to express costimulatory molecules such as CD86. The expression level of CD86 on the three main types of OVA^+^ myeloid cells was examined on day 2 after injection. MigDCs had the highest level of CD86, followed by LTDCs, and then monocytes and macrophages (Fig. 6a).

After injection of adjuvanted vaccines, MigDCs in the draining lymph node can be further divided into three subpopulations, namely migratory CD8α^+^ DCs that are CD11b^−^CD8^+^Ly6C^−^, migratory CD11b^+^ DCs that are CD11b^+^CD8^−^Ly6C^−^, and monocytes-derived Monocyte-derived dendritic cells (MoDCs) that are CD11b^+^Ly6C^+17^. The majority of OVA was in CD11b^+^ DCs while some OVA was in MoDCs following injection of Nano-11 adjuvanted OVA. Among OVA-negative MigDCs, more CD8α^+^ type DCs and fewer MoDCs were present (Fig. 6b). This suggests that Nano-11 is preferentially taken up by CD11b^+^ DCs and MoDCs.

### Nano-11 retains antigen at the injection site

OVA-containing cells appeared in the draining lymph node for a shorter period of time after injection of soluble OVA compared with adjuvanted OVA. We hypothesized that absorption of antigen onto AH or Nano-11 helped retain it at the injection site and prolonged the supply of antigen to the draining lymph node. To test this, we utilized whole mouse imaging again to assess how long Alexa Fluor-647 labeled OVA stayed at the injection site with different formulations (Fig. 6c). Immediately after immunization, OVA was readily detected at the injection site with all three formulations. One day after immunization soluble OVA was present at a lower level compared with the adjuvanted formulations. However, by day 2 soluble OVA was barely detectable at the injection site, corresponding to the rapid decrease in the lymph node on day 2, while the presence of adjuvanted OVA remained high. On day 7, no soluble OVA was detected at the injection site but there was still residual OVA when formulated with AH or Nano-11. This confirmed that Nano-11 retained antigen at the injection site at a comparable level as AH for the first 7 days following injection.

## Discussion

Nanotechnology holds great potential for the development of improved vaccine adjuvants and delivery systems. In order to develop nanoparticles as safe and effective adjuvants, it is important to understand the fate and biodistribution of the nanoparticles in vivo.^12^ The interaction of nanoparticles with cells, extracellular matrix and plasma proteins depends on their size, shape, surface charge, and chemical composition.^19^ We recently reported that Nano-11, positively charged alpha-D-glucan dendrimer nanoparticles, enhances the immune response when combined with different protein antigens.^16^ In vitro experiments with bone marrow-derived DCs demonstrated that Nano-11 is readily phagocytized and enhances the uptake of adsorbed antigens. In addition, Nano-11 induced activation of DCs as indicated by increased expression of costimulatory molecules CD80 and CD86, and secretion of IL-1β.^16^ In the current series of experiments, we assessed the effect of Nano-11 on DCs in vivo and the biodistribution following intramuscular injection. The results indicate that Nano-11 prolongs the retention of antigen at the injection site; induces a transient inflammatory response characterized by the recruitment of macrophages and relatively few neutrophils; facilitates the uptake and transport of antigen by MigDCs to the draining lymph node; and does not distribute systemically to internal organs.

Vaccine adjuvants act locally at the site of injection and should enhance the transport of vaccine antigens to the draining lymph node where the adaptive immune response is initiated. It is preferable that the adjuvant does not reach other organs and tissues to avoid systemic effects. Assessment of the distribution of fluorochrome-labeled Nano-11 indicated that the adjuvant remains largely localized to the site of injection. A small amount of the adjuvant reached the draining lymph nodes as demonstrated by imaging of excised lymph nodes and analysis of DCs and macrophages by flow cytometry. There was no evidence of accumulation of Nano-11 in other organs including the lungs, liver, and kidneys. The fluorescent signal at the injection site decreased rapidly during the first day and was higher on day 2 after injection. The lower level of fluorescence on day 1 compared with day 2 may be the result of edema associated with the acute local inflammatory response to the injected nanoparticles. The increased extracellular fluid absorbs some of the fluorescent signals leading to a transient decrease of the fluorescent signal. The fluorescence gradually decreased over time indicating that the nanoparticles disappeared from the injection site. Most of the nanoparticles were taken up by macrophages accumulating at the site of injection.

Nano-11 is positively charged and adsorbs negatively charged protein antigens such as OVA similar to positively charged AH.^16^ Imaging of fluorescently labeled OVA indicated that soluble OVA rapidly disappears from the injection site, but adsorption to either aluminum hydroxide adjuvant or Nano-11 retains the antigen at the injection site for several days. This is consistent with earlier studies on aluminum adjuvants.^20^ The retention increases the period of time during which antigen is available for phagocytosis by APCs that are recruited to the injection site and may be important for the adjuvant effect of Nano-11.

Adjuvants often trigger a local inflammatory response at the site of injection as a result of direct activation of inflammatory cells and indirectly by the mechanical trauma associated with the injection of particulate material or emulsions. The inflammation allows the recruitment of APCs to the injection site where they can take up the vaccine antigens (“reverse targeting”). Previous studies showed that Nano-11 induces the secretion of IL-1β by DCs in a caspase-1-dependent fashion.^16^ IL-1β is a potent pro-inflammatory stimulus and this indicates that Nano-11 has the intrinsic property to induce an inflammatory response. Similar to Nano-11, aluminum adjuvants induce IL-1β secretion by DCs.^21–23^ However, the inflammatory response induced by aluminum adjuvanted vaccines is characterized by an early infiltration of many neutrophils in comparison with Nano-11 (ref. 24). Neutrophils accumulate quickly in response to cell injury and can further contribute to tissue damage through the release of proteolytic enzymes and reactive oxygen species.^25, 26^ It has been suggested that neutrophil accumulation is detrimental to the immune response to vaccines because they compete for vaccine antigens with APCs,^27^ although depletion of neutrophils prior to vaccination did not affect the antibody response to aluminum adjuvanted antigens.^24^ Nevertheless, administration of antigens with Nano-11 may induce less tissue damage and increase the availability of antigens for APCs in comparison with aluminum adjuvants. The limited tissue damage induced by Nano-11 may also contribute to the diminishment of the inflammation after day 2 and a substantial decrease of inflammatory cell number at the injection site by day 21. This is in contrast to the long lasting granulomatous inflammation that can be induced by aluminum adjuvants, although such granulomas are rarely associated with clinical symptoms.^28^

Following injection of fluorochrome-labeled Nano-11, fluorescent cells appeared in the draining lymph node on day 1, increased in number on day 2, and had nearly disappeared on day 7. The majority of Nano-11-positive cells were identified as MigDCs suggesting that the cells picked up Nano-11 in tissues and migrated to the lymph node. The uptake of particulate material by DCs is influenced by the size, shape, surface charge, and chemical composition.^12^ Smaller nanoparticles are preferentially taken up by DCs, whereas microparticles are preferentially phagocytized by macrophages.^29, 30^ Cationic particles are more efficiently taken up than neutral charged or anionic particles.^30^ Nanoparticles can enhance the delivery of antigens to lymph nodes by increasing phagocytosis by MigDCs. Assessment of OVA-positive APCs in the lymph node following injection of soluble OVA or combined with either Nano-11 or aluminum adjuvant showed that both adjuvants increase the number of OVA-containing APCs in the lymph nodes. However, Nano-11 induced a greater uptake of OVA in comparison with aluminum adjuvant on a per cell basis. There are several explanations for the increased efficiency of the delivery of OVA to the lymph node by Nano-11. First, aluminum hydroxide adjuvant forms large aggregates, about 17 µm in size, which are not easily phagocytized in contrast to the 70–80 nm Nano-11 nanoparticles. Secondly, as discussed above, the accumulation of neutrophils at the injection site induced by aluminum adjuvants may interfere with antigen uptake by DCs. Thirdly, Nano-11 provides a stronger maturation stimulus to DCs as indicated by the increased expression of costimulatory molecules, which may enhance the migration to the draining lymph node. Following injection of Nano-11 with OVA, most of the OVA-containing cells were MigDCs with high expression of CD86 and they were comprised predominantly of CD11b^+^ DCs and moDCs. These subpopulations of DCs are highly effective in inducing the adaptive immune response.^31–33^

In summary, we have demonstrated that positively charged *α*-D-glucan nanoparticles (Nano-11) induce a local inflammatory response at the injection site and effectively target antigen to MigDCs. The inflammation resolved within 2 to 3 weeks and there was no evidence of long-term deposition or systemic distribution of the nanoparticles. These findings support the further development of Nano-11 as a safe and effective vaccine adjuvant.

## Methods

### Mice and immunization

Six-week-old female BALB/c mice were purchased from Harlan, Inc. (Indianapolis, IN) and housed with food and water ad libitum. All procedures were conducted in accordance with the Guide for the Care and Use of Laboratory Animals of the National Institutes of Health, and approved by the Purdue University Animal Care and Use Committee. For comparison of the immune response to different doses of adjuvants, mice were randomly assigned to experimental groups with six mice per group. The group size was based on estimates of expected variation in antibody titers to identify significant differences between experimental groups with a power of 80%. The investigators were not blinded to the analysis. Mice were injected in both calf muscles with a volume of 50 μL each containing 20 μg/mL rPA (List Biological, Campbell, CA) alone or with 2 mg/mL, 500 μg/mL, or 125 μg/mL Nano-11 or AH (Rehydragel, General Chemical, Berkeley Heights, NJ). Antigen and adjuvant were mixed by end-over-end rotation in 1.5 mL microcentrifuge tubes for 1 h prior to injection. Three weeks after the first injection, mice were boosted with the same formulation. Two weeks after the booster injection, mice were euthanized and serum samples were collected. For all the other experiments, mice were immunized with 100 µg/mL Alexa Fluor 647-labeled OVA (Sigma, St. Louis, MO) or OVA (Invivogen, San Diego, CA) with or without 2 mg/mL Alexa Fluor 647-labeled or nonlabeled Nano-11, or 2 mg/mL AH in 2 mM Tris-saline buffer (pH 7.4) and mice were euthanized at indicated time points.

### Enzyme-linked immunosorbent assay (ELISA)

Ninety-six well plates were coated with 1 μg/mL rPA overnight at 4 °C. The plates were washed five times with 0.05% Tween in phosphate-buffered saline (PBS), blocked with 200 μL 1% bovine serum albumin for 1 h, and 100 μL serial dilutions of the serum samples were added to the wells in duplicates. After another hour of incubation, plates were washed again and incubated with 100 μl of peroxidase-conjugated goat anti-mouse IgG (#AP124P; Sigma) or peroxidase-conjugated goat anti-mouse IgG1 (#1073-05; Southern Biotech, Birmingham, AL) or IgG2a (#1080-05; Southern Biotech) for 1 h. After the final wash, 100 μL TMB substrate solution (Sigma) was added to the wells and the plates were kept in dark at room temperature for 20 min. Reaction was stopped with 50 μL 2 M sulfuric acid and the fluorescence absorbance at 450 nm (OD 450) was measured in a microplate reader (BioTEK, Winooski, VT). Titers were calculated as the dilution at which the OD 450 reached 0.05.

### Preparation of Nano-11 and Alexa Fluor 647-labeled Nano-11

Nano-11 was prepared as described previously.^16^ To label Nano-11 with Alexa Fluor 647 (ThermoFisher, Waltham, MA), 1 g of Nano-11 was first acidified in 6 mL of 2.5 M HCl for 30 min with constant agitation. Excessive acid was initially washed by the addition of 20 mL of a 90% isopropanol solution (v/v) followed by constant agitation for 2 h. This mixture was then centrifuged at 3000×*g* for 10 min and the supernatant was decanted. Further washing was performed by repetitive resuspension in new isopropanol solution, agitation for 20 min, and centrifugation. The sample was washed two more times after the decanted supernatant tested negative for chloride ions (i.e., no observation of AgCl white haze) upon the drop-wise addition of 0.1 M AgNO_3_.Thereafter, the dry acidified Nano-11 powder was obtained by rinsing the mixture two times using 100% ethanol, constant agitation for 1 h, centrifugation, and grinding. After that, 12 mg of acidified Nano-11 was dissolved in 600 µL of 50 mM solution of 2-(*N*-morpholino) ethanesulfonic acid (MES) buffer. Twelve micrograms of 1-ethyl-3-(3-dimethylamino-propyl)carbodiimide (EDAC) was dissolved in 1.2 mL of MES buffer and then added to the Nano-11 dispersion. One microgram of Alexa Fluor 647 was dissolved in 1 mL of MES buffer and an aliquot of 120 µL was added immediately after EDAC and Nano-11 were mixed. This mixture was then incubated under constant agitation for 4 h at room temperature and 5 h at 4 °C. After the labeling, unbound Alexa Fluor 647 dye was separated from labeled Nano-11 through centrifugal infiltration with Nanosep (Pall Co, Port Washington, NY) at 12,000×*g* for 20 min followed by additional seven times of wash with MES buffer containing 100 mM trizma base. Alexa Fluor 647-labeled Nano-11 was finally purified with three rounds of dialysis wash (3, 3, and18 h) with 10 K MWCO Slide-A-Lyzer™ G2 dialysis cassettes (ThermoFisher, Waltham, MA) in MES buffer containing 2 ppm NaN_3._ The final product was stored at 4 °C for future use.

### In vivo imaging

One day before in vivo imaging, mice were shaved and treated with Nair to remove hair from the lower part of the body. After immunization mice were imaged on an IVIS Lumina II In Vivo Imaging System (PerkinElmer, Waltham, MA). Mice were anesthetized through isoflurane inhalation during imaging and fluorescent signals in the images were analyzed by Living Image software (PerkinElmer, Waltham, MA).

### Immunofluorescence

At the indicated times after immunization, mice were euthanized by CO_2_ asphyxiation and the injection site samples were excised. The tissues were fixed in 4% paraformaldehyde for 3 h, dehydrated in 30% sucrose solution overnight, embedded in optimal cutting temperature medium, snap frozen in liquid nitrogen and stored at −80 °C. Eight micrometer thick sections of the tissue were obtained with a Leica CM1860 cryostat(Leica Biosystem, Buffalo Grove, IL). Sections were stained with the following antibodies (all from Biolegend, San Diego, CA): Biotinylated anti-I-A/I-E (#107603; APCs), anti-Ly-6G (#127603; neutrophils), anti-Mac-2 (#125403; macrophages), anti-Ly-6C (#128003; monocytes). The antibodies were detected with Alexa Fluor 488-conjugated streptavidin (Jackson Immunoresearch Laboratory, West Grove, PA). Eosinophils were stained with phenol red.^34^ Sections were then embedded in Prolong Gold antifading with 4',6-diamidino-2-phenylindole (Invitrogen, Carlsbad, CA), coverslipped, sealed, and stored at 4 °C. The number of fluorescently labeled cells at the injection site was counted within high-power field on a fluorescence microscope (Nikon E400). Confocal images of the injection sites were obtained with a Nikon A1R MP microscope (Nikon).

### Flow cytometry

Iliac lymph nodes were excised from immunized mice and immediately immersed in Hank's Balanced Salt Solution medium and put on ice. Lymph nodes were then treated with 1 mg/mL Collagenase D (Roche Diagnostics, Indianapolis, IN) at 37 °C for 20 min to release cells. Cells were washed with PBS containing 2 mM EDTA, blocked with 1% normal rabbit serum, and stained with the following antibodies and reagents: Zombie Violet, biotinylated anti-Ly6G, biotinylated anti-Ly6C, Alexa Fluor 488 or PerCP-Cy5.5-conjugated anti-CD11c, PE-conjugated anti-I-A/I-E, PE/Cy7-conjugated anti-CD86, Alexa Fluor 488-conjugated F4/80 (all from BioLegend, San Diego, CA), and PE-conjugated anti-Siglec F (BD Bioscience, San Jose, CA). Biotinylated antibodies were detected with Alexa Fluor 488 or APC-conjugated streptavidin. After staining, cells were fixed in 2% paraformaldehyde. Cells were examined in a Canto II flow cytometer (BD Bioscience, San Jose, CA), and data were analyzed with FlowJo software (FlowJo, Eugene, OR).

### Statistical analysis

The bars in the graphs represent the mean ± SEM. For antibody titer comparison, titers were first log transformed and the difference between different formulations was then determined with one-way analysis of variance (ANOVA) with Tukey’s post hoc analysis. For all other experiments, the statistical significance of differences between different groups was determined with one-way ANOVA with Newman-Keuls post hoc analysis for multiple group comparison or Student’s test for two group analysis. Differences were considered significant when *p* < 0.05.

## Electronic supplementary material

Supplementary Information
