# Supplementary material for: Alpha-D-glucan nanoparticulate adjuvant induces a transient inflammatory response at the injection site and targets antigen to migratory dendritic cells
Source: NPJ Vaccines. 2017 Feb 23;2:4. doi: 10.1038/s41541-017-0007-7 (PMC5627279; doi:10.1038/s41541-017-0007-7)
Supplement: Supplementary file 1 — Supplementary Information [file 41541_2017_7_MOESM1_ESM.pdf]

Supporting Information

Alpha-D-glucan Nanoparticulate Adjuvant Induces a Transient Inflammatory Response at the Injection Site and Targets Antigen to Migratory Dendritic Cells.

Fangjia Lu, Yung-Yi Mosley, Randol J Rodriguez Rosales, Brooke Carmichael, Srikanth Elesela, Yuan Yao, Harm HogenEsch[*]

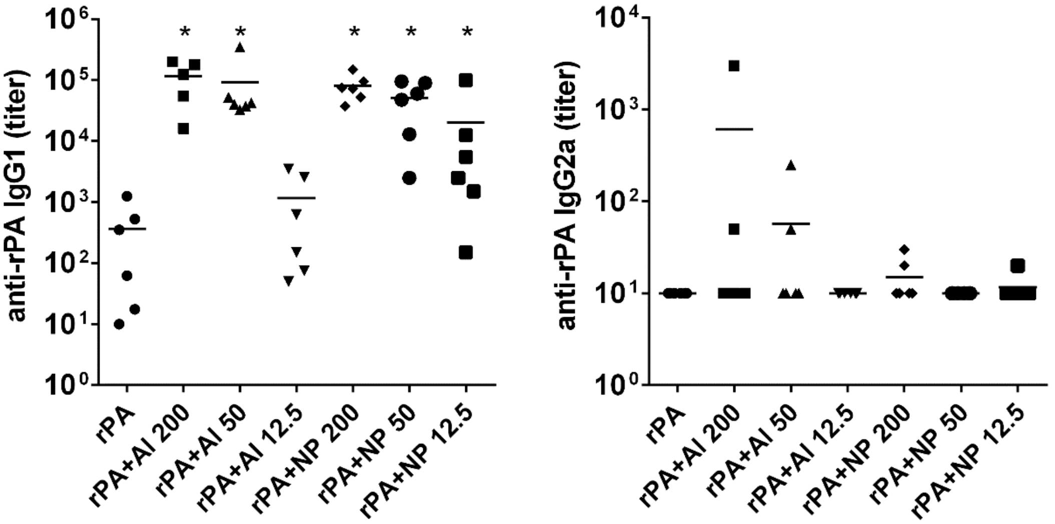

Supplemental Figure 1. Titers for rPA-specific IgG1 and IgG2a antibodies in serum after two injections of 2 µg recombinant protective antigen (rPA) from *Bacillus anthracis* alone or combined with either AH or Nano-11 at three different dosages (200 µg, 50 µg, and 12.5 µg). Titers were measured by ELISA. Each symbol represents a mouse and the bar indicates the geometric mean. * p<0.05 vs. rPA alone.

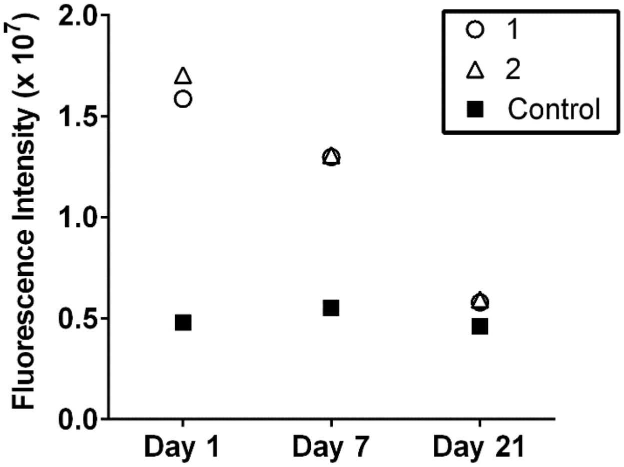

Supplemental Figure 2. Low levels of fluorescence in the draining (iliac) lymph node on Days 1, 7 and 21 after intramuscular injection of 200 µg Alexa Fluor 647-labeled Nano-11 (mouse 1 and 2) or nonlabeled Nano-11 (Control) with 10 µg OVA. Mice were euthanized at Day 1, 7, and 21 after injection, and the iliac lymph nodes were imaged.

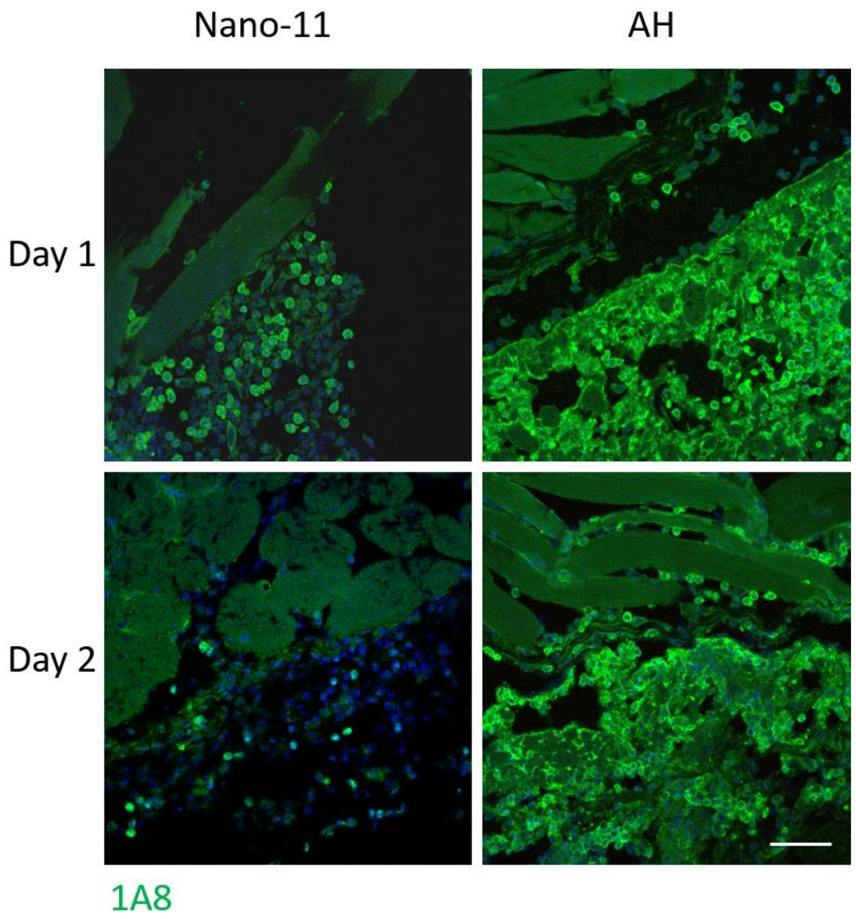

Supplemental Figure 3. Neutrophils at the injection sites on Day 1 and Day 2 for either Nano-11 or AH injected mice. Confocal images were taken under a 40X objective lens on a confocal microscope and are representative of four independent experiments. Bar=50µm

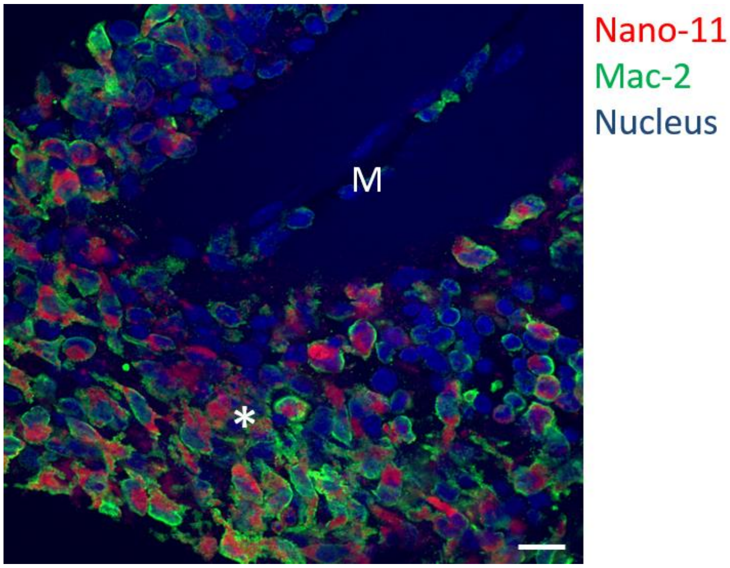

Supplemental Figure 4. Most Nano-11 at the injection site is inside Mac-2$^+$ cells by Day 2. Confocal images were taken under a 60X objective lens and are representative of two independent experiments. M: muscle, *: injection site. Bar=20µm.

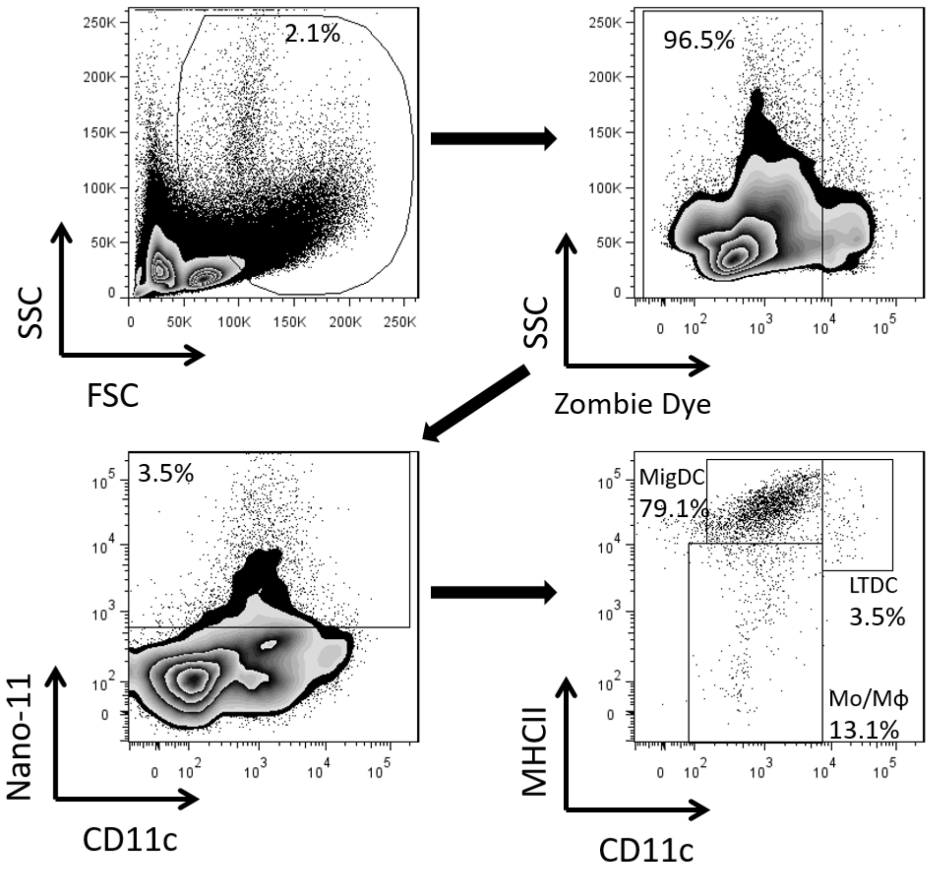

Supplemental Figure 5. Gating strategy for detecting and phenotyping Nano-11-containing myeloid cells in the draining lymph nodes demonstrated on a lymph node sample harvested on Day 2. Representative of three independent experiments is shown here.

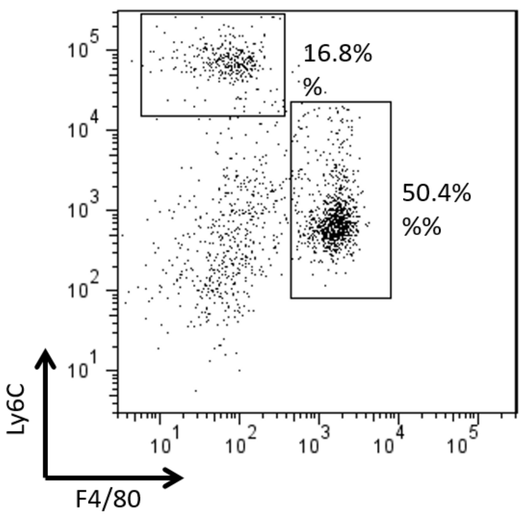

Supplemental Figure 6. Phenotyping for the MHCII$^{low\text{-}medium}$CD11c^{medium} population in the draining lymph node. Representative of two independent experiments is shown here.

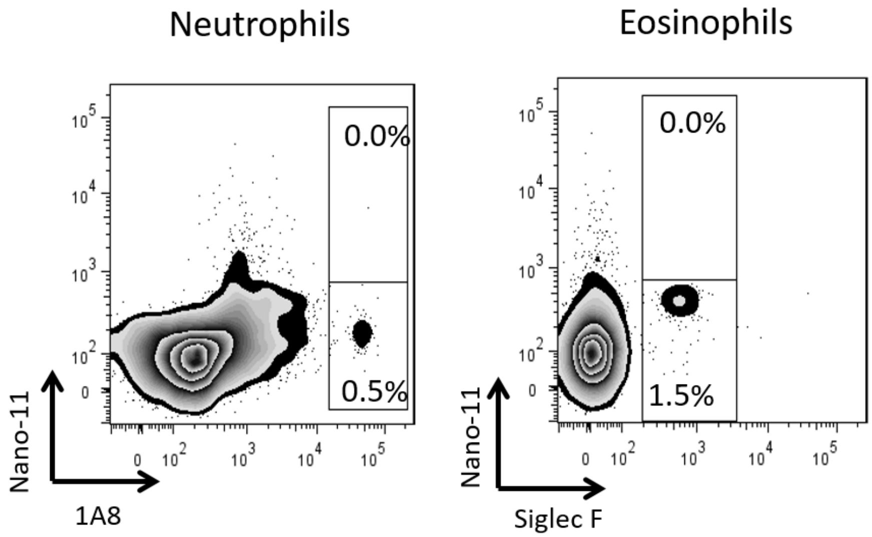

Supplemental Figure 7. Absence of Nano-11 from neutrophils and eosinophils in the draining lymph node. Data were obtained one day after immunization and are representative of two independent experiments.
